# The ethical PROMISE NFR dataset: data for ethical concern classification in software requirements

**DOI:** 10.1016/j.dib.2026.113366

**Published:** 2026-10-21

**Authors:** Vijaylaxmi Bittal, Soni Sweta

**Affiliations:** Department of Computer Engineering, Mukesh Patel School of Technology Management & Engineering, SVKM’s Narsee Monjee Institute on Management Studies (NMIMS) Deemed-to-University, Mumbai, 400056, India

**Keywords:** Ethical requirements, Requirement engineering, AI ethics, Software Engineering Datasets

## Abstract

The Ethical PROMISE NFR dataset is an extension of the widely utilized PROMISE Non-Functional Requirements (NFR) dataset annotated using a multi-label ethical annotation framework comprising nine ethical concern categories: transparency, accountability, fairness, bias, privacy, accessibility, user consent, sustainability, and human autonomy. The dataset contains 3677 software requirement statements collected from the PROMISE repository and manually tagged using a concern-based ethical taxonomy considering responsible AI principles, the General Data Protection Regulation (GDPR) and the European Union AI Act. To assess annotation reliability, the dataset was independently reviewed by two annotators. Direct inter-annotator agreement between Annotator 1 and Annotator 2 yielded Cohen’s κ = 0.6164 with 80.11% observed agreement. Against the reference annotations, Annotator 1 achieved κ = 0.9093 and Annotator 2 achieved κ = 0.5585, with an average agreement of 86.21%. The dataset is publicly available on IEEE DataPort and Mendeley Data and can support research in ethical requirements engineering, responsible AI, software requirements classification, explainable artificial intelligence, and natural language processing. The data may also be used to develop, train, and evaluate machine learning and deep learning models for ethical concern detection in software requirements.

Specifications Table

**Table:** 

Subject	Computer Sciences
Specific subject area	Software Engineering, Requirement Engineering, AI Ethics
Type of data	Table,Text,.csv,.xlsx
Data collection	Software requirements were taken from the PROMISE NFR dataset. Ethical concern labels were manually assigned using a concern-based annotation by referring AI principles, GDPR, and the European Union AI Act.
Data source location	Original PROMISE-NFR Dataset: https://data.mendeley.com/datasets/4ysx9fyzv4/1 Newly Annotated Ethical PROMISE NFR Dataset: Mendeley Data: doi:10.17632/hrd9btkbsz.2 IEEE DataPort: doi:10.21227/0esx-c487
Data accessibility	Repository name: Ethical PROMISE NFR Dataset Data identification number: doi:10.17632/hrd9btkbsz.2 Direct URL to data: https://data.mendeley.com/datasets/hrd9btkbsz/2 Data identification number: 10.21227/0esx-c487 Direct URL to data: https://ieee-dataport.org/documents/ethical-promise-nfr-dataset Instructions: This dataset contains two types of labels: a functional label and a multi-label ethical annotation. The functional label retains the functional and non-functional classifications from the PROMISE NFR Dataset. The ethical annotation consists of three ethical concern mappings selected from nine predefined ethical categories and represented using the three levels Severe, Moderate, and Mild according to their relative ethical relevance to the requirement.
Related research article	None

## 1 Value of the data

• Ethical PROMISE NFR is extended and enhanced version of PROMISE NFR dataset.
• The multi-label annotated dataset consists of 9 ethical concern categories,
  Privacy, Fairness, Transparency, Accountability, User Consent, Accessibility
  Sustainability, Human Autonomy and Bias.
• Researchers in software engineering, requirements engineering, responsible AI, natural language processing, and explainable AI can use the dataset to develop, train, and evaluate machine learning and deep learning models for ethical concern classification.
• The dataset and accompanying annotation process may be reused and adapted for annotating ethical concerns in other software requirements datasets, facilitating future research on trustworthy and ethically aligned software systems.

## 2 Background

With the increasing use of AI systems, some of the ethical issues [1] that need to be considered include privacy, fairness, transparency etc. The majorly used data sets for requirements classifications are illustrated in Table 1 below.

**Table 1:** Existing datasets.

Sl No	Dataset name	Label/Annotations	Link
1	FR_NFR Dataset	- Functional Requirement - Non-Functional Requirement [1,2]	https://data.mendeley.com/datasets/4ysx9fyzv4/1
2	Promise+Dataset	- F,A,L,LF,MN,O,PE,SC,SE,US,FT,PO [3]	https://zenodo.org/records/12805484
3	Promise NFR Dataset	- F=Functional, A=Availability, L = Legal, LF = Look and feel, MN = Maintainability, O = Operational, PE = Performance, SC = Scalability, SE = Security, US = Usability, FT = Fault tolerance, and PO = Portability [4]	https://zenodo.org/records/268542

In Table 1, Table 3 different datasets having their unique label types, based on whether they are functional or non-functional. In Promise NFR Dataset, non-functional requirements can be seen under labels such as A, L, LF, MN, O, PE, SC, SE, US, FT, and PO. However, the absence of any specific label has been found related to ethical requirements.

User-generated feedback, such as app reviews, has also been studied as a useful source of software requirements and software engineering information [5]. However, such sources do not provide a structured ethical annotation framework for requirement-level analysis.

The European Union Artificial Intelligence Act, which entered into force in 2024, establishes requirements for the development and use of AI systems, particularly those classified as high-risk [6]. These requirements emphasize areas such as risk management, transparency, human oversight, accountability, and protection of fundamental rights. Related responsible-AI considerations are also reflected in data-protection requirements under the GDPR [3] and transparency-oriented documentation practices such as Model Cards [4]. Although the PROMISE NFR Dataset provides a comprehensive framework for functional and non-functional requirements, it does not include dedicated labels for ethical concerns. The absence of such annotations limits its direct use for studying ethical considerations relevant to responsible AI and regulatory frameworks such as the EU AI Act [6]. The Ethical PROMISE NFR Dataset addresses this gap by introducing structured ethical concern annotations.

## 3 Data Description

Ethical PROMISE NFR Dataset is a repository containing two files: annotated dataset and README document. The annotated dataset includes 3677 software requirement statements from the PROMISE NFR dataset that have been annotated using nine ethical concern categories: Privacy, Fairness, Accountability, Transparency, Human Autonomy, Accessibility, Bias, User Consent, and Sustainability. Each requirement is assigned three ethical concern mappings represented using the levels Severe, Moderate, and Mild according to their relative ethical relevance to the requirement. Each data record has six attributes: ProjectID, RequirementText, functional_label, Ethical_label, text_cleaned, and ethical_reason. The ethical_reason attribute provides the rationale for the assigned multi-label ethical mappings and supports transparency and explainability of the annotation process. The README document explains all the details of the dataset including description of attributes, ethical concerns' descriptions, annotation process, validation, and how to use the dataset. Together, these files facilitate reproducibility, interpretation, and reuse of the dataset for research in ethical requirements engineering, responsible AI, natural language processing, and machine learning*.* Table 2 shows summary of Ethical Promise NFR Dataset.

**Table 2:** Summary of ethical PROMISE NFR dataset.

Attribute	Description
Dataset Name	Ethical PROMISE NFR Dataset
Source Dataset	PROMISE NFR Dataset
Number of Requirements	3677
Annotation Type	Manual multi-label concern-based ethical annotation
Ethical Categories	Privacy, Fairness, Accountability, Transparency, Human Autonomy, Accessibility, Bias, User Consent, Sustainability
Number of Ethical Categories	9
Ethical Mappings per Requirement	3
Mapping Levels	Severe, Moderate, Mild
Dataset Attributes	ProjectID, RequirementText, functional_label, Ethical_label, text_cleaned, ethical_reason
Validation Method	Two independent annotators; percentage agreement and Cohen’s Kappa
**Inter-Annotator Agreement (IAA)**	**80.11%**
**Inter-Annotator Cohen’s Kappa**	**Cohen’s κ = 0.6164**
Annotator 1–Reference Agreement	κ = 0.9093; Agreement = 95.30%
Annotator 2–Reference Agreement	κ = 0.5585; Agreement = 77.11%
Average Annotator-to-Reference Agreement	86.21%
Repository Contents	Annotated dataset file and README documentation
Data Format	CSV/XLSX
Repository	IEEE DataPort and Mendeley Data
DOI	IEEE DataPort: 10.21227/0esx-c487; Mendeley Data: 10.17632/hrd9btkbsz.2

Distributions of ethical concern categories shown in Table 3.

**Table 3:** Distributions of ethical concern categories.

Ethical concern	Frequency	Percentage (%)
Human Autonomy	2170	19.67
Transparency	2169	19.66
Privacy	1583	14.35
Bias	1557	14.11
Accountability	1525	13.82
Accessibility	694	6.29
User Consent	619	5.61
Fairness	544	4.93
Sustainability	170	1.54
**Total**	**11,031**	**100.00**

The annotation framework assigns Severe, Moderate, and Mild mappings to each requirement according to relative ethical relevance. Across the 3677 requirements, this results in 3677 Severe mappings, 3677 Moderate mappings, and 3677 Mild mappings, representing 33.33% of the 11,031 total ethical mappings at each mapping level.

The most frequent ethical concern co-occurrences included Human Autonomy with Transparency, Bias with Human Autonomy, Accountability with Transparency, Bias with Transparency, and Privacy with Transparency. Complete pairwise co-occurrence statistics are provided in Supplementary File S6.

### 3.1 Description of the ethical_reason attribute

The ethical_reason attribute captures the reason behind the labeling of ethical concerns for each software requirement. The attribute is helpful in providing a clear justification for classifying requirements under different ethical concerns categories. Instead of being yet another label, this attribute helps capture the reasoning behind the manual annotation. For example, a requirement labeled under Privacy could have its ethical_reason captured as “This requirement is about safeguarding users' personal data from any unauthorized access.” The attribute may also help in building XAI models since it captures the justification behind the ethical classification.

Representative examples of annotated requirements are shown in Table 4.

**Table 4:** Representative examples of annotated requirements.

Requirement statement	Ethical concern	Ethical reason
The system shall provide a history report of changes made to the Activity or Event data.	**Privacy – Severe, User Consent – Moderate, Human Autonomy – Mild**	Privacy is the primary ethical mapping because the requirement involves collection, processing, storage, filtering, or presentation of data. User Consent is the secondary mapping because informed permission and choice may be relevant, while Human Autonomy is contextually related to user control over data-related interactions.
The product shall ensure that it can only be accessed by authorized users. The product will be able to distinguish between authorized and unauthorized users in all access attempts.	**Privacy – Severe, Transparency – Moderate, Accountability – Mild**	Privacy is the primary ethical mapping because the requirement concerns protection from unauthorized access. Transparency is secondarily relevant to the visibility and understandability of access decisions, while Accountability is contextually relevant to responsibility and traceability of system access.
The application shall ask the user to confirm dropping a player or trading a player before performing the action.	**User Consent – Severe, Human Autonomy – Moderate, Transparency – Mild**	User Consent is the primary ethical mapping because the system explicitly requires confirmation before an action is performed. Human Autonomy is secondarily relevant because the user retains control over the decision, while Transparency is contextually relevant because confirmation makes the intended action visible before execution.

## 4 Experimental Design, Materials and Methods

### 4.1 Data source

Ethical PROMISE NFR Dataset was developed based on PROMISE Non-Functional Requirement (NFR) Dataset, which acts as the source dataset. PROMISE NFR dataset comprises of the statements of software requirements collected from various software engineering projects and is frequently utilized in researches in the domain of requirements engineering [7]. The requirement statements were extracted and prepared for ethical concern annotation.

### 4.2 Concern-based ethical taxonomy

As depicted in Table 5 below, an ethical taxonomy based on concerns was created in order to aid the annotation procedure. This taxonomy is made up of nine ethical concerns namely: Transparency, Accountability, Fairness, Bias, Privacy, Accessibility, User Consent, Sustainability, and Human Autonomy. These concerns have been identified through the analysis of the ethical principles and regulations for responsible AI, such as the GDPR and EU AI Act. The rules and guidelines for the annotation procedure were set forth for each concern category.

**Table 5:** Concern-based ethical taxonomy.

Category	Description	Related laws/Frameworks	Representative requirement
Transparency	The degree to which the system’s operations, decisions, and data usage are explainable and understandable to users and stakeholders.	GDPR [3], EU AI Act [6]	Logging during migration may be turned on or off at any time.
Human Autonomy	The extent to which the system preserves user control and avoids manipulation or over-reliance.	EU AI Act [6]	If a lead is scored highly, the system will supply lead data and score information.
Accountability	The ability to trace responsibility for system decisions and actions.	GDPR [3], EU AI Act [6]	The ELSS must only permit deletion messages to be generated under defined conditions.
Fairness	The extent to which the system ensures equitable treatment across different user groups.	EU AI Act [6]	The Disputes System shall accurately provide a list of possible reason codes.
Bias	The presence and mitigation of systematic errors favoring certain groups over others.	EU AI Act [6]	The system shall be able to infer incidents from probe vehicle data.
Privacy	Protection of user data and limitation of unnecessary data collection and processing.	GDPR [3]	The system must collect and process context-specific data for users.
Accessibility	The usability of the system by people with diverse abilities and disabilities.	WCAG [8], ADA [9]	Management processes shall be accessible with screen-reading and magnification software.
User Consent	The extent to which users are informed and give explicit permission for data usage.	GDPR [3]	Customers will be able to create accounts to store their customer profiles.
Sustainability	The extent to which the system supports environmentally responsible resource use, efficient operation, and responsible long-term lifecycle behavior. Technical maintainability or availability alone does not constitute ethical Sustainability unless the requirement also reflects broader resource, environmental, or lifecycle-related implications.	EU Green Deal [10]	**The estimator shall apply selected recycled parts to the collision estimate.**

Note: The representative requirements illustrate how the proposed concern-based taxonomy and multi-label mapping approach were applied during annotation. Ethical Sustainability is distinguished from conventional technical NFR concepts such as maintainability and availability. Requirements concerning only technical uptime, maintenance, or operational performance are not treated as Sustainability on that basis alone; Sustainability is applied where broader resource-efficiency, environmental, or long-term lifecycle implications are also present.

The proposed taxonomy complements existing ethical and responsible-AI frameworks by applying nine ethical concern categories to individual software requirement statements. The framework supports multi-label annotation and records the relative relevance of the selected concerns using Severe, Moderate, and Mild mappings.

### 4.3 Annotation procedure

Each requirement statement was manually reviewed against the nine predefined ethical concern categories and assigned three ethical-category mappings according to the annotation guidelines. The annotation process followed a concern-oriented approach in which requirements were analyzed for the presence of ethical implications related to the predefined concern categories. The three selected categories were ranked as Severe, Moderate, and Mild according to their relative ethical relevance to the requirement. Severe represents the strongest or primary ethical mapping, Moderate represents the secondary ethical mapping, and Mild represents a contextual or comparatively weaker ethical mapping. The three mappings assigned to each requirement represent three distinct ethical categories; therefore, the same ethical category is not assigned at more than one level within the same requirement. The mapping order is meaningful and reflects decreasing relative ethical relevance from Severe to Moderate to Mild. Where no direct match was identified, the nearest contextually relevant ethical categories were assigned. In addition to the ethical labels, a brief rationale describing the reason for label assignment was recorded to improve transparency and support future explainability studies.

Where a requirement was relevant to more than one ethical concern, the annotators assessed each concern against the predefined category definitions and ranked the three most relevant concerns according to their relative ethical relevance. Borderline cases were identified where more than one category could reasonably apply at a similar level or where the distinction between adjacent mapping levels was unclear. Such cases were reviewed using the annotation rubric, the requirement context, and the recorded ethical rationale, and were resolved through consensus between Annotator 1 and Annotator 2.

### 4.4 Annotation workflow

The annotation was carried out based on a defined workflow to ensure consistency and repeatability. First, the requirements were analyzed according to the PROMISE NFR Dataset. Formatting errors were corrected while retaining the original semantics. Two independent annotators then reviewed the requirements using the ethical concern taxonomy and the predefined annotation guidelines. Each requirement was assessed against the nine ethical concern categories and assigned three ethical-category mappings represented as Severe, Moderate, and Mild according to their relative ethical relevance. Where a direct ethical match was not evident, the nearest contextually relevant categories were selected. During annotation, the annotators referred to the category definitions, examples, and criteria provided in the annotation guidelines. The annotations produced by Annotator 1 and Annotator 2 were subsequently compared using percentage agreement and Cohen’s Kappa to assess annotation reliability. Disagreement cases were reviewed and resolved through consensus between Annotator 1 and Annotator 2 using the annotation rubric. The entire process is described in detail in Supplementary File S2 and S3.

### 4.5 Annotator qualifications and training

The annotation process was carried out by two industry experts who have expertise in the areas of software engineering and digital technologies. The qualifications of the two annotators and their areas of expertise are shown in Table 6. Prior to carrying out the annotation exercise, both the annotators had been introduced to the taxonomy of the ethical concern, annotation criteria, and examples through an annotation process. The annotation exercise was piloted to ensure consistent understanding of the categories of ethical concerns prior to the validation exercise. The pre-interview protocol used for sample collection is given in Supplementary File S1

**Table 6:** Annotator qualifications.

Annotator	Designation	Relevant expertise
Annotator 1	Manager, Website & SEO	Experience in digital platforms, web-based systems, and user-oriented requirements
Annotator 2	Lead Software Engineer	Experience in software development, software engineering practices, and requirement-related activities

### 4.6 Annotation guidelines

Each software requirement was manually assigned multiple ethical concern mappings according to the concern-based annotation guidelines. The criteria followed during annotation are summarized in Table 7.

**Table 7:** Annotation guidelines.

Guideline	Description
Annotation type	Manual
Label assignment	Multi-label
Annotation basis	Relative ethical relevance to the requirement
Mapping levels	Severe – strongest or primary ethical relevance; Moderate – clear secondary ethical relevance; Mild – contextual or comparatively weaker ethical relevance
Number of ethical categories	9
Ethical frameworks	GDPR, EU AI Act, WCAG, ADA, EU Green Deal
Validation	Two independent annotators using Cohen’s Kappa and percentage agreement
Severity disagreement handling	Differences in the assigned mapping level for the same ethical category were treated as disagreements and were reviewed by Annotator 1 and Annotator 2 using the annotation rubric. Final assignments were resolved through consensus**.**
Disagreement resolution	Disagreement cases were reviewed and resolved through consensus between Annotator 1 and Annotator 2 using the annotation rubric

### 4.7 Annotation validation

Annotation reliability was evaluated across the complete set of 3677 software requirements. Two annotators independently reviewed all requirements using the predefined ethical taxonomy and annotation guidelines. Inter-annotator agreement and reliability were assessed using percentage agreement and Cohen’s Kappa coefficient (κ).

For inter-annotator reliability analysis, the unit of analysis was each requirement–ethical-category pair. Each of the 3677 requirements was evaluated separately across the nine ethical concern categories, resulting in 33,093 paired category-level annotation decisions. For each category, the annotation assigned by Annotator 1 and Annotator 2 was represented by one of four mutually exclusive values: Severe, Moderate, Mild, or Nil, where Nil indicated that the category was not assigned to that requirement. Exact agreement required both annotators to assign the same value to the same ethical category. Differences between Severe, Moderate, Mild, and Nil were treated as disagreements, and no partial-agreement weighting was applied.

The reference annotations correspond to the original ethical category-level annotations recorded in the validation dataset before the independent reviews by Annotator 1 and Annotator 2. These reference annotations are distinct from the independent annotations subsequently provided by Annotator 1 and Annotator 2 and from the final reconciled annotations produced after disagreement resolution. Against the reference annotations, Annotator 1 achieved an observed agreement of 95.30% with Cohen’s κ = 0.9093, while Annotator 2 achieved an observed agreement of 77.11% with Cohen’s κ = 0.5585. The average annotator-to-reference agreement was 86.21%. These annotator-to-reference comparisons are reported separately and are not interpreted as inter-annotator reliability. Direct comparison between Annotator 1 and Annotator 2 resulted in an Inter-Annotator Agreement (IAA) of 80.11% and Cohen’s κ = 0.6164, indicating substantial agreement according to Landis and Koch [11]. Differences between the two annotators may reflect ambiguity in requirements involving overlapping ethical concerns and differences in assigning relative relevance levels.

Disagreement cases were subsequently reviewed and resolved through consensus between Annotator 1 and Annotator 2 using the annotation rubric. Following reconciliation of the disagreement cases, final label consistency was approximately 94.07%, with Cohen’s κ = 0.8855. This post-reconciliation value represents the consistency of the finalized annotations after consensus-based reconciliation and is not interpreted as an independent measure of inter-annotator reliability. Category-wise pre- and post-reconciliation results are presented in Table 8. Detailed Cohen’s Kappa calculations, including observed agreement (Po), expected agreement (Pe), annotator-to-reference comparisons, direct inter-annotator comparisons, and disagreement analysis, are provided in Supplementary Files S2 and S3.

(1) k=(Po−Pe)/(1−Pe)

where,

• k = Cohen’s Kappa coefficient
• Po = Observed agreement between annotators
• Pe = Expected agreement by chance

**Table 8:** Category-wise annotation agreement before reconciliation and final label consistency after reconciliation.

Concern category	Pre-reconciliation κ	Interpretation	Post-reconciliation final label consistency κ	Interpretation
Transparency	0.6357	Substantial	0.8940	Almost Perfect
Human Autonomy	0.6330	Substantial	0.8890	Almost Perfect
Accountability	0.6278	Substantial	0.8902	Almost Perfect
Fairness	0.5403	Moderate	0.8657	Almost Perfect
Bias	0.6310	Substantial	0.8859	Almost Perfect
Privacy	0.6201	Substantial	0.8833	Almost Perfect
Accessibility	0.5402	Moderate	0.8448	Almost Perfect
User Consent	0.5496	Moderate	0.8576	Almost Perfect
Sustainability	0.4019	Moderate	0.7732	Substantial
**Average**	**0.5755**	**Moderate**	**0.8649**	**Almost Perfect**

**Note:** Cohen’s Kappa values were interpreted according to Landis and Koch [11]: ≤0.20 (Slight), 0.21–0.40 (Fair), 0.41–0.60 (Moderate), 0.61–0.80 (Substantial), and 0.81–1.00 (Almost Perfect Agreement).

### 4.8 Dataset construction

Following validation of the annotation process, all 3677 software requirement statements in the PROMISE NFR dataset were manually annotated using the proposed concern-based taxonomy. The Ethical PROMISE NFR Dataset consists of 3677 requirement statements with the associated ethical concerns and metadata. The final dataset incorporates the reconciled annotations obtained after disagreement cases were reviewed and resolved by Annotator 1 and Annotator 2. The dataset is freely accessible through IEEE DataPort and Mendeley Data and can be applied to various fields such as ethical requirements engineering, ethical concern detection, natural language processing, machine learning, and explainable AI.

## Limitations

The Ethical PROMISE NFR Dataset is an extension of the PROMISE NFR dataset and thus possesses similar properties to its source data. Although the dataset uses a multi-label annotation approach, ethical interpretation may still involve a degree of subjectivity, and alternative classifications of some requirements may be possible. The Severe, Moderate, and Mild levels indicate the relative ethical relevance of the assigned concerns and should not be interpreted solely as the magnitude of ethical harm or risk. The annotation reliability was evaluated using two independent annotators. Direct comparison between Annotator 1 and Annotator 2 resulted in Cohen’s κ = 0.6164 with 80.11% observed agreement, indicating substantial agreement. Against the reference annotations, Annotator 1 achieved κ = 0.9093 and Annotator 2 achieved κ = 0.5585. Disagreement cases were subsequently reviewed and resolved through consensus between the two annotators. Following reconciliation, final label consistency was approximately 94.07%, with Cohen’s κ = 0.8855.

The distribution of ethical concern mappings is imbalanced, with some categories, particularly Sustainability, occurring less frequently than others. Researchers using the dataset for machine-learning applications should therefore consider suitable class-imbalance handling strategies such as class weighting, stratified evaluation, resampling, or data augmentation depending on the downstream task.

For future machine-learning applications using this dataset, researchers may consider evaluation measures suitable for multi-label and imbalanced classification, including micro- and macro-averaged precision, recall, and F1-score, together with per-category performance measures.

Future extensions may include additional ethical concern categories, validation with a larger number of annotators, and application of the annotation framework to other software requirements datasets.

## Ethics Statement

The authors have read and followed the ethical requirements for publication in Data in Brief and confirm that the current work does not involve human subjects, animal experiments, or any data collected from social media platforms.

## CRediT Author Statement

**Vijaylaxmi Bittal:** Conceptualization, Methodology, Validity tests, Data curation, Writing- Original draft preparation. **Soni Sweta:** Supervision, Reviewing and Editing.

## Appendix Supplementary materials

Image, application 1

Image, application 2

Image, application 3

Image, application 4

Image, application 5

Image, application 6
