# Supplementary material for: The ethical PROMISE NFR dataset: data for ethical concern classification in software requirements
Source: Data Brief. 2026 Oct 21;69:113366. doi: 10.1016/j.dib.2026.113366 (PMC13733468; doi:10.1016/j.dib.2026.113366)
Supplement: Supplementary file 1 [file mmc1.docx]

**ANNOTATOR INTERVIEW PROTOCOL**

**Ethical Requirement Annotation in the PROMISE NFR Dataset**

*Semi-Structured Interview and Pilot Assessment Instrument*

| **Researcher** | Vijaylaxmi Bittal |
| --- | --- |
| **Programme** | PhD Scholar, Computer Engineering, MPSTME,NMIMS Mumbai |
| **Research Guide** | Dr. Soni Sweta |
| **Study Focus** | Identification and classification of ethical concerns in software requirements |
| **Instrument Purpose** | Selection and readiness assessment of annotators |

*Confidential research instrument*

# 1. Purpose of the Interview

This interview is designed to assess whether prospective annotators have sufficient understanding of software requirements, ethical concerns, severity levels, independent annotation procedures, and the use of operational definitions. The interview should be conducted before the formal annotation of the PROMISE NFR dataset.

# 2. Interview Administration Instructions

The interviewer should ask the questions in the order presented, while allowing reasonable follow-up probes. Responses should be recorded using an anonymised Annotator ID. The interviewer should not train or correct the candidate during the interview. Training may be provided after the selection decision. The pilot annotation should be completed independently and should not be included in the final research dataset.

# 3. Ethical and Confidentiality Statement

Participation is voluntary. Responses will be used only for research-related selection, training, and quality assurance. Personal identifiers should not be included in the research dataset. Annotators should be informed that they may withdraw before the formal annotation begins without penalty.

# 4. Candidate Information

| **Annotator ID** |  |
| --- | --- |
| **Date of Interview** |  |
| **Mode of Interview** |  |
| **Educational Qualification** |  |
| **Current Role / Experience** |  |
| **Relevant Domain Experience** |  |
| **Previous Annotation Experience** |  |
| **Interviewer Name** |  |

# 5. Pre-Interview Consent Checklist

- ☐ Yes	☐ No	The purpose of the interview has been explained.
- ☐ Yes	☐ No	The candidate understands that participation is voluntary.
- ☐ Yes	☐ No	The candidate agrees to the recording of written interview notes.
- ☐ Yes	☐ No	The candidate understands the confidentiality arrangements.
- ☐ Yes	☐ No	The candidate agrees to complete an independent pilot annotation task.

# A. Background and Eligibility

**1. Please briefly describe your educational and professional background.**

Response: __________________________________________________________________________________

___________________________________________________________________________________________

**2. Do you have experience in software engineering, requirements engineering, artificial intelligence, data privacy, responsible AI, software testing, or natural language processing? Please specify.**

Response: __________________________________________________________________________________

___________________________________________________________________________________________

**3. Have you previously worked with software requirements documents or requirement datasets?**

Response: __________________________________________________________________________________

___________________________________________________________________________________________

**4. Are you familiar with functional and non-functional requirements?**

Response: __________________________________________________________________________________

___________________________________________________________________________________________

**5. How would you explain the difference between a functional requirement and a non-functional requirement?**

Response: __________________________________________________________________________________

___________________________________________________________________________________________

**6. Have you previously participated in a data annotation, coding, classification, or content-analysis task?**

Response: __________________________________________________________________________________

___________________________________________________________________________________________

**7. Are you familiar with ethical concerns in software or AI-based systems?**

Response: __________________________________________________________________________________

___________________________________________________________________________________________

**8. Are you familiar with standards or regulations such as GDPR, the EU AI Act, WCAG, or ADA?**

Response: __________________________________________________________________________________

___________________________________________________________________________________________

# B. Understanding of the Research Task

**9. In your own words, what is an ethical requirement in a software system?**

Response: __________________________________________________________________________________

___________________________________________________________________________________________

**10. Do you believe that one software requirement can contain more than one ethical concern? Please explain with an example.**

Response: __________________________________________________________________________________

___________________________________________________________________________________________

**11. Can a technically correct requirement still create an ethical concern? Why?**

Response: __________________________________________________________________________________

___________________________________________________________________________________________

**12. How would you distinguish between a requirement that creates an ethical risk and a requirement that protects users from an ethical risk?**

Response: __________________________________________________________________________________

___________________________________________________________________________________________

**13. What would you do when an ethical concern is implied rather than explicitly stated?**

Response: __________________________________________________________________________________

___________________________________________________________________________________________

**14. Should an annotator rely only on the written requirement, or also make assumptions about the wider system context? Explain.**

Response: __________________________________________________________________________________

___________________________________________________________________________________________

# C. Understanding of Ethical Categories

**15. What does transparency mean in the context of a software requirement?**

Response: __________________________________________________________________________________

___________________________________________________________________________________________

**16. Would the requirement “The system shall generate recommendations without explaining the factors used” involve transparency? Why?**

Response: __________________________________________________________________________________

___________________________________________________________________________________________

**17. What does human autonomy mean in an automated system?**

Response: __________________________________________________________________________________

___________________________________________________________________________________________

**18. What ethical concern may arise when users cannot override an automated decision?**

Response: __________________________________________________________________________________

___________________________________________________________________________________________

**19. How is accountability different from transparency?**

Response: __________________________________________________________________________________

___________________________________________________________________________________________

**20. Would logging system decisions automatically make a system accountable? Explain.**

Response: __________________________________________________________________________________

___________________________________________________________________________________________

**21. How would you distinguish between fairness and bias?**

Response: __________________________________________________________________________________

___________________________________________________________________________________________

**22. Consider: “The system shall rank candidates using historical recruitment data.” Which ethical categories might apply, and why?**

Response: __________________________________________________________________________________

___________________________________________________________________________________________

**23. Can a requirement involve bias without explicitly mentioning demographic groups?**

Response: __________________________________________________________________________________

___________________________________________________________________________________________

**24. Can a system be unbiased at the data level but still produce unfair outcomes?**

Response: __________________________________________________________________________________

___________________________________________________________________________________________

**25. How would you distinguish between privacy and user consent?**

Response: __________________________________________________________________________________

___________________________________________________________________________________________

**26. Consider: “The system shall collect users’ location data continuously.” What additional information would you look for before assigning privacy or consent labels?**

Response: __________________________________________________________________________________

___________________________________________________________________________________________

**27. If users provide consent, does that automatically remove all privacy concerns? Why or why not?**

Response: __________________________________________________________________________________

___________________________________________________________________________________________

**28. What types of requirements would you classify under accessibility?**

Response: __________________________________________________________________________________

___________________________________________________________________________________________

**29. Would multilingual support be considered an accessibility concern? Please justify your answer.**

Response: __________________________________________________________________________________

___________________________________________________________________________________________

**30. What does sustainability mean in relation to software and AI systems?**

Response: __________________________________________________________________________________

___________________________________________________________________________________________

**31. Would high computational resource usage always be classified as a sustainability concern? Explain.**

Response: __________________________________________________________________________________

___________________________________________________________________________________________

# D. Severity Assessment

**32. How would you distinguish between low, medium, and high ethical severity?**

Response: __________________________________________________________________________________

___________________________________________________________________________________________

**33. Which factors should influence severity assessment, such as type of data, number of users affected, reversibility of harm, legal consequences, discriminatory impact, vulnerability, user control, duration, or frequency?**

Response: __________________________________________________________________________________

___________________________________________________________________________________________

**34. Consider: “The system shall store user passwords in plain text.” Which ethical category and severity level would you assign? Explain.**

Response: __________________________________________________________________________________

___________________________________________________________________________________________

**35. Consider: “The system shall collect anonymous usage statistics.” Would you assign an ethical category? What severity would you select?**

Response: __________________________________________________________________________________

___________________________________________________________________________________________

**36. How would you annotate a positive ethical safeguard such as “The system shall encrypt all personal data”?**

Response: __________________________________________________________________________________

___________________________________________________________________________________________

**37. Should a positive ethical requirement be assigned no concern, low severity, or a separate positive-practice label? Explain your choice.**

Response: __________________________________________________________________________________

___________________________________________________________________________________________

# E. Handling Ambiguity and Annotation Discipline

**38. What would you do if a requirement could belong to two or more ethical categories?**

Response: __________________________________________________________________________________

___________________________________________________________________________________________

**39. What would you do if you were unsure whether a category applies?**

Response: __________________________________________________________________________________

___________________________________________________________________________________________

**40. Would you select all potentially relevant categories, only clearly supported categories, leave the requirement unlabelled, or add an uncertainty note? Explain.**

Response: __________________________________________________________________________________

___________________________________________________________________________________________

**41. How would you handle a vague requirement such as “The system shall handle user information appropriately”?**

Response: __________________________________________________________________________________

___________________________________________________________________________________________

**42. Should annotators discuss difficult requirements with each other during independent annotation? Why?**

Response: __________________________________________________________________________________

___________________________________________________________________________________________

**43. Would you be comfortable recording a short justification for every selected ethical category?**

Response: __________________________________________________________________________________

___________________________________________________________________________________________

**44. How much context should an annotator infer when a requirement contains limited information?**

Response: __________________________________________________________________________________

___________________________________________________________________________________________

**45. What would you do if your personal ethical opinion differs from the operational definition in the annotation guideline?**

Response: __________________________________________________________________________________

___________________________________________________________________________________________

# F. Annotation Process and Reliability

**46. Are you comfortable assigning binary labels where 1 means the ethical category is present and 0 means it is absent?**

Response: __________________________________________________________________________________

___________________________________________________________________________________________

**47. Are you comfortable assigning multiple ethical categories to a single requirement?**

Response: __________________________________________________________________________________

___________________________________________________________________________________________

**48. Can you complete the annotation independently without consulting another annotator?**

Response: __________________________________________________________________________________

___________________________________________________________________________________________

**49. Are you willing to follow the same annotation guideline throughout the study?**

Response: __________________________________________________________________________________

___________________________________________________________________________________________

**50. Are you comfortable revisiting disputed annotations after the inter-annotator agreement analysis?**

Response: __________________________________________________________________________________

___________________________________________________________________________________________

**51. How would you maintain consistency when annotating a large dataset?**

Response: __________________________________________________________________________________

___________________________________________________________________________________________

**52. What type of examples or training would help you perform the annotation accurately?**

Response: __________________________________________________________________________________

___________________________________________________________________________________________

**53. How much time would you need to annotate approximately 50, 100, and 500 requirements?**

Response: __________________________________________________________________________________

___________________________________________________________________________________________

**54. Are you able to complete the annotation without using external AI tools unless their use is explicitly permitted by the researcher?**

Response: __________________________________________________________________________________

___________________________________________________________________________________________

# G. Final Readiness Questions

**55. Which ethical categories do you find easiest to understand?**

Response: __________________________________________________________________________________

___________________________________________________________________________________________

**56. Which ethical categories appear most difficult or overlapping?**

Response: __________________________________________________________________________________

___________________________________________________________________________________________

**57. Is the difference between fairness and bias sufficiently clear?**

Response: __________________________________________________________________________________

___________________________________________________________________________________________

**58. Is the difference between privacy and user consent sufficiently clear?**

Response: __________________________________________________________________________________

___________________________________________________________________________________________

**59. Is the difference between transparency and accountability sufficiently clear?**

Response: __________________________________________________________________________________

___________________________________________________________________________________________

**60. Are the severity definitions clear enough for consistent annotation?**

Response: __________________________________________________________________________________

___________________________________________________________________________________________

**61. What changes would you recommend in the annotation guideline?**

Response: __________________________________________________________________________________

___________________________________________________________________________________________

**62. Do you feel confident that you can independently annotate the requirements using the provided guideline?**

Response: __________________________________________________________________________________

___________________________________________________________________________________________

# 6. Pilot Scenario-Based Assessment

Ask the candidate to annotate each scenario independently. For each scenario, record the selected category or categories, severity level, and justification.

## Scenario 1

*The system shall automatically share customer information with third-party service providers.*

- Which ethical categories apply?
- What severity would you assign?
- Which words influenced your decision?

Candidate annotation: ____________________________________________________________________

Justification: ____________________________________________________________________________

## Scenario 2

*The system shall allow an administrator to reverse an automated decision.*

- Which ethical category applies?
- Does this create an ethical risk or reduce one?

Candidate annotation: ____________________________________________________________________

Justification: ____________________________________________________________________________

## Scenario 3

*The system shall use previous loan repayment data to determine customer eligibility.*

- Could fairness or bias apply?
- What additional context would be useful?
- Would you annotate a potential or implicit concern?

Candidate annotation: ____________________________________________________________________

Justification: ____________________________________________________________________________

## Scenario 4

*The system shall record all user activity for security purposes.*

- Does the stated purpose remove privacy concerns?
- Would consent or transparency also apply?
- How would you determine severity?

Candidate annotation: ____________________________________________________________________

Justification: ____________________________________________________________________________

## Scenario 5

*The application shall support keyboard navigation and screen readers.*

- Which ethical category applies?
- Is this an ethical concern or an ethical safeguard?

Candidate annotation: ____________________________________________________________________

Justification: ____________________________________________________________________________

# 7. Interview and Pilot Scoring Rubric

| **Criterion** | **Maximum Score** | **Score Awarded** |
| --- | --- | --- |
| Understanding of software requirements | 0–2 |  |
| Understanding of ethical categories | 0–3 |  |
| Ability to distinguish overlapping categories | 0–3 |  |
| Severity assessment | 0–2 |  |
| Quality of justification | 0–2 |  |
| Consistency across similar requirements | 0–3 |  |
| Ability to avoid unsupported assumptions | 0–2 |  |
| **Total** | **17** |  |

# 8. Selection Guidance

| **Score Range** | **Interpretation** |
| --- | --- |
| 14–17 | Suitable with minimal training |
| 10–13 | Suitable after additional training |
| Below 10 | Not recommended for the main annotation round |

# 9. Interviewer Decision Record

Overall assessment: ☐ Suitable	☐ Suitable after training	☐ Not suitable

Training required: ☐ No	☐ Yes

Recommended role: ☐ Primary annotator	☐ Pilot annotator only	☐ Reserve annotator

Key strengths: ____________________________________________________________________________

Areas requiring training: __________________________________________________________________

Final comments: ____________________________________________________________________________

Interviewer signature: ______________________________ Date: ______________________________
